# Effects of growth factor deprivation on MKN-45 spheroid cells

**DOI:** 10.55730/1300-0152.2646

**Published:** 2023-01-18

**Authors:** Özlem TÜRKSOY TERZİOĞLU, Gökhan TERZİOĞLU

**Affiliations:** 1 University of Health Sciences, Hamidiye Institute of Health Sciences, Department of Molecular Biology and Genetics, İstanbul, Turkey; 2 University of Health Sciences, Hamidiye International School of Medicine, Department of Medical Biology, İstanbul, Turkey; 3 University of Health Sciences, Validebağ Research Center, İstanbul, Turkey

**Keywords:** Gastric cancer, serum deprivation, growth factor deprivation, epidermal growth factor, fibroblast growth factor, fetal bovine serum, spheroid formation, cancer stem cell

## Abstract

**Background/aim:**

Serum and growth factor deprivation, a common cellular stressor in solid tumors, arises upon irradiation, chemotherapy, and antiangiogenesis. Spheroid body culture aims to enrich cancer stem cells by using low attachment conditions and some growth factors, such as basic fibroblast growth factor and epidermal growth factor to support the spheroid formation in serum-free spheroid culture. However, spheroid culture without any growth factors can imitate the tumor environment more realistically.

In this study, we aimed to identify the effect of growth factor deprivation on the MKN-45 gastric cancer cell line in terms of stemness characteristics.

**Materials and methods:**

The spheroids were obtained by culturing MKN-45 gastric cancer cells in low attachment conditions, and then spheroids were dissociated to obtain cells for further analyses. Self-renewal, multipotency, cellular transformation, invasiveness, chemoresistance, and the expression of stemness-related genes were analyzed using tumor spheroid formation assay, soft agar colony formation assay, transwell invasion assay, chemosensitivity assay, and quantitative RT-PCR assay, respectively.

**Results:**

Fetal bovine serum and growth factor deprivation caused an increase in stemness markers of OCT4, NANOG, SOX2, MUC1, CD24 and CD90. Increasing functional aggressiveness-related properties, such as self-renewal, chemoresistance, and invasive ability, have also been observed in fetal bovine serum-growth factor-free conditions.

**Conclusion:**

Growth factors may not be essential for spheroid culture to enrich cancer stem cells. The deprivation of both fetal bovine serum and growth factors also induces a more aggressive phenotype in MKN-45 cells; thus, it provides an opportunity for further studies targeting tumor cells.

## 1. Introduction

Gastric cancer, the fifth most common cancer, is mainly treated with surgery, chemotherapy, and radiotherapy (Smyth et al., 2020). It is usually diagnosed at late stages and has a recurrence rate of 70% despite successful surgery. Thus, it is still the third leading cause of mortality among all cancer types (Eusebi et al., 2020). A subpopulation of gastric cancer cells, cancer stem cells (CSCs), probably have major roles in the initiation, progression, and recurrence of cancer (Addeo et al., 2021). CSCs have broad capabilities, including high self-renewal, chemoresistance, proliferation, and metabolic modification (Yang et al., 2020). Therefore, CSCs are figured as important targets for the treatment of gastric cancer. Even though many methods have been utilized to obtain cancer stem cells, these methods are mostly expensive and inefficient. Finding an efficient method for the enrichment of cancer stem cells is a priority.

3D cell culture is a better approach to culture cells for maintenance and recovery of primary phenotype when compared to 2D cell culture (Fennema et al., 2013). Multicellular spheroid culture is also more suitable for mimicking the environment and growth conditions of tumors under in vitro conditions; furthermore, expression levels of stemness-related markers increase in spheroid forming cells (Kapałczyńska et al., 2018). CSCs are enrichable and obtainable using the spheroid culture system (Chen et al., 2012). In this system, a serum-free medium and mitogens, such as the epidermal growth factor (EGF) and basic fibroblast growth factor (bFGF or FGF-2), are generally used (Zhong et al., 2010; Ma et al., 2019). However, this method needs several weeks of incubation, and using the mitogens is not an economical approach.

The MKN-45 cell line was established from a liver metastasis of a poorly differentiated adenocarcinoma of the stomach. MKN-45 cells are capable of forming spheroid bodies with cancer stem cell properties, and there are several studies on spheroid body formation in these cells (Liu et al., 2013). Some growth factors (GFs) and supplements, such as FGF-2 and EGF, are used to support the spheroid formation in serum-free spheroid culture of MKN-45 cells (Hajimoradi et al., 2016). However, most liver metastases of gastric cancer become hypovascular due to the depletion of nutrients and growth factors, including basic fibroblast growth factor (Namasivayam et al., 2007). This phenomenon also occurs in colorectal cancer, hepatocellular carcinoma, and liver metastasis of colorectal cancer compared to adjacent tissues (Mathonnet et al., 2006). Therefore, spheroid culture should be performed without any growth factors to simulate tumor environment more realistically. The MKN-45 cell line is tolerant to nutrient deprivation, so it is suitable to study the effects of GFs and serum starvation in spheroid culture (Izuishi et al., 2000).

We analyzed the effects of the deprivation of growth factors and serum on MKN-45 gastric cancer cells via analyzing the changes in the expression levels of stemness genes, chemoresistance, and invasiveness in spheroids. We acquired spheroid bodies using two kinds of serum-free mediums with or without growth factors to evaluate cell characteristics, such as self-renewal and stem cell-related markers. This study analyzed the expression changes in Octamer-binding transcription factor 4 (OCT4), Nanog homeobox (NANOG), SRY-Box Transcription Factor 2 (SOX2), Cluster of Differentiation 24 (CD24), Cluster of Differentiation 44 (CD44), Cluster of Differentiation 90 (CD90), and Mucin short variant S1 (MUC1) genes, as candidate markers for gastric cancer stem cells, chemoresistance, spheroid formation, and invasiveness (Nishii et al., 2009; Takaishi et al., 2009; Zhang et al., 2011; Jiang et al., 2012; Saeki et al., 2014; Terzioğlu et al., 2018). There were increased expression levels for most of these stemness markers in the spheroids cultured in the fetal bovine serum (FBS) and GF-free medium compared to other groups. Self-renewal capacity, chemoresistance, and invasiveness were also higher in the spheroid cells compared to parental cells. However, especially spheroids cultured with no GF had more elevated levels of them. This finding suggests that GF depletion induces stemness and aggressiveness; therefore, GFs may not be essential for spheroid formation in vitro.

## 2. Materials and methods

### 2.1. Cell culture

MKN-45 cells were maintained in RPMI-1640 medium (Gibco, USA) containing 10% FBS (Gibco, USA) and plated at a density of 1 × 10^6^ live cells in T75 flasks.

Spheroid bodies were derived by plating 2 × 10^6^ parental cells in T75 flasks coated with 2% agarose and 0.9% NaCl. The first experimental group (FBS− GF−) was cultured in RPMI-1640 medium with 100 units/mL penicillin and 100 g/mL streptomycin (Thermo Fisher Scientific, USA), without FBS and GFs. The other experimental group (FBS− GF+) was cultured in the same medium with the addition of 20 ng/mL human FGF-2 and 20 ng/mL EGF (BD Biosciences, USA) (Table 1). After the fifth day of the culture, spheroid bodies were counted under an inverted microscope (Leica, Germany) at 100× magnification followed by dissociation using Acutase (Millipore) and reseeding. Five days later after reseeding, we analyzed the secondary spheroids.

### 2.2. Quantitative real-time PCR

Total RNA was isolated from cultured cells using the RNEasy kit (Qiagen, Germany) and subsequently reverse transcribed to complementary DNA (cDNA) via reverse transcription by Quantitech Reverse Transcription Kit (Qiagen, Germany). Quantitative real-time PCR (qRT-PCR) was performed using predesigned TaqMan gene expression assays (Thermofisher Scientific, USA). Each sample was assayed in triplicate. The changes in the expression levels of stemness markers, OCT4, NANOG, SOX2, MUC1, CD24, and CD90, were analyzed. The housekeeping gene for normalization was beta-actin (ACTB). The expression changes in the target genes were calculated using the ΔΔCt method. The list of TaqMan gene expression assays is shown in Table 2.

### 2.3. Soft agar colony formation assay

Soft agar colony formation assays were performed to determine the cellular transformation ability of spheroid cells under anchorage-independent conditions. Each well of a six-well plate was coated with 1 mL of RPMI-1640 medium with 10% FBS and 1% agarose. After 20 min of incubation at 37° C, the bottom layer solidified, and then 1 mL of RPMI-1640 medium with 10% FBS and 0.7% agarose containing 500 spheroid cells or adherent cells (upper layer) was added on it. The plates were incubated at 37° C, 5% CO_2_. The nutrition was replenished by adding 150 −L of RPMI-1640 medium with 10% FBS every 3 days for 3 weeks. After 21 days in culture, the plates were stained using 0.05% crystal violet for colony quantification. Colonies with more than 50 cells were counted under an inverted light microscope.

### 2.4. In vitro cell invasion assay

Transwells (Millicell) were coated with 100 −L of matrigel for 30 min at 37 °C and then used to determine the invasiveness of both the spheroids and parental cells. Cell suspension of dissociated spheroids was seeded into the upper chambers at the density of 2 × 10^5^ cells. Each upper chamber contained a 200-−L serum-free medium while each lower chamber contained a 750-−L RPMI-1640 medium containing 10% FBS. After 24 h of incubation, the membranes were fixed with 3.7% formaldehyde for 2 min. They were permeabilized with 100% methanol for 20 min and then stained with Giemsa for 15 min. Cells that did not invade but remained on the membrane of each upper chamber were scraped by a cotton swab. Invading cells were visualized and counted in four different fields under an inverted microscope at 400× magnification.

### 2.5. Chemosensitivity assay

5-Fluorouracil (5-FU) was used for chemosensitivity assay due to its common use in gastric cancer therapy (Xu et al., 2015). The minimum drug concentration was determined according to the dose which is lethal to 50% of parental cells (LC50). The drug concentrations are compatible with the literature (Imada et al., 1997; Cho et al., 2002) Spheroid-derived cells and parental cells were seeded in 96-well plates at a density of 2000 cells/well in 150 −L of RPMI-1640 medium (3 wells per group) with or without 5-Fluorouracil (5-FU) (Kocak). For the chemosensitivity assay, the cells were treated with 1–2 −g/mL of 5-FU for 48 h. MTS assay was used to determine the relative survival fraction of cells. One Solution Reagent (Promega) was added to each well at the 48th hour of 5-FU treatment and incubated at 37 °C for 2 h. Cell viability was assessed according to the absorbance measurements at 490 nm with an ELx800 ELISA microplate reader (BioTek).

### 2.6. Flow cytometry

Spheroid bodies were washed once with phosphate-buffered saline (PBS), dissociated using Acutase, and centrifuged to obtain cell pellets. Cells were resuspended, filtered, and stained with a CD44-specific antibody (Biolegend 103026). The experiments were conducted using BD FACSAria III (Becton Dickinson, USA). The quadrants in the flow cytometric analysis software were adjusted according to the unstained samples.

### 2.7. Statistical analysis

The Kruskal–Wallis and Mann–Whitney *U* tests were used for statistical analysis. The differences were considered statistically significant at p < 0.05 (indicated as ‘*’ for Kruskal–Wallis, ‘#’ for Mann–Whitney *U* test).

## 3. Results

### 3.1. FBS− GF− medium enhances spheroid body formation in MKN-45 cells

**Figure 1:**
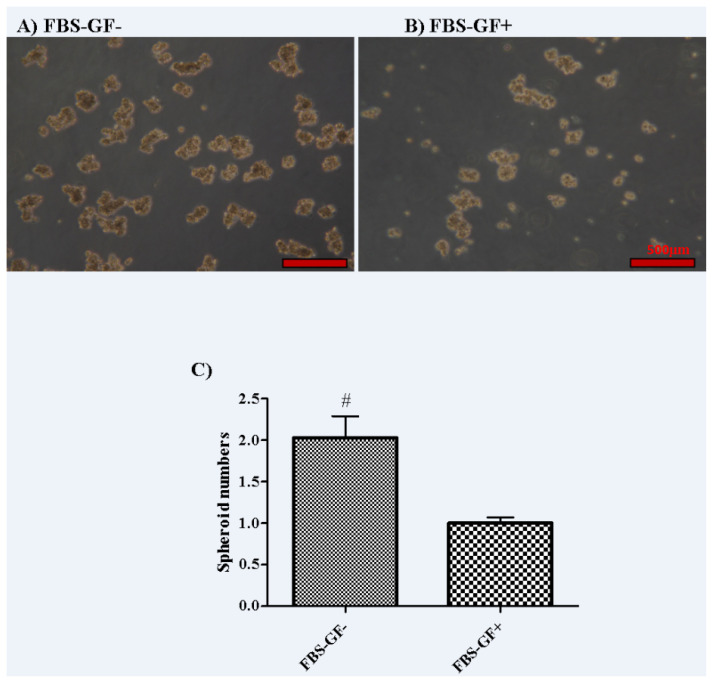
Comparison of the effect of growth factors (GFs), FBS-positive and GF–FBS-free conditions on spheroid-forming activity in MKN45 cell line. After 10 days, at 100× magnification. A) Group FBS− GF−, B) Group FBS− GF+, C) Fold change of spheroid numbers over Group FBS− GF+. Data are presented as the means ± SD. Comparison of spheroid-forming activity. [‘#’ indicates a statistically significant difference compared to Group FBS− GF+ (p < 0.05)]

There is no significant difference between the groups of FBS− GF− and FBS− GF+ in terms of the size and viability of spheroids. Therefore, the data is not shown. However, the group of FBS− GF− had a 2 times higher number of spheroids when compared to the group of FBS− GF+ (p = 0.04) (Figure 1).

### 3.2. Cells grown in FBS− GF− medium exhibit higher stem cell marker levels than cells in FBS− GF+ medium

**Figure 2:**
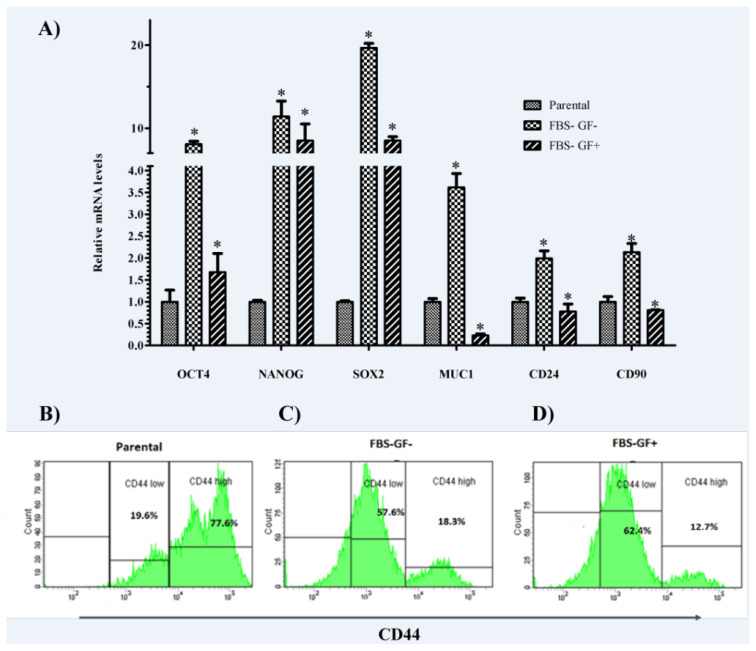
Stem cell-related genes were upregulated in spheroid derived cells over parental counterparts A) CSC marker gene expression levels, flow cytometry analysis of CD44− CD44^low^ and CD44^high^ cells: B) Parental, C) Group FBS− GF−, D) Group FBS− GF+. “*” indicates a statistically significant difference between parental and spheroid groups (p < 0.05).

Expression changes in OCT4, NANOG, SOX2, MUC1, CD24, and CD90 genes were analyzed by qRT-PCR to monitor whether spheroid bodies grown in different culture systems cause enrichment of cells expressing stem cell markers (Figure 2). The results indicated that MKN-45 spheroids from two different culture systems showed different expression patterns of stem cell markers.

The addition of the GFs significantly decreased the expression of OCT4, MUC1, NANOG, SOX2, CD24, and CD90 (p = 0.02, p = 0.0003, p = 0.0006, p = 0.03, p = 0.001, and p = 0.001, respectively). Gene expression changes in spheroid cells are shown in Figure 2.

### 3.3. Expression of CD44 decreases in FBS free conditions

CD44 levels were analyzed using a flow cytometer (Figures 2B–2D). The ratio of CD44 positive cells decreased in spheroids compared to parental cells. The parental cells also had a larger CD44 high population (77.6%). (Figures 2C, 2F, 2G). The FBS− GF− group had an elevated level of CD44 high expression (18.3%) (Figure 2C) compared to the group of FBS− GF+ (12.7%) (Figure 2D).

### 3.4. FBS− GF− medium enhances colony formation capacity

**Figure 3:**
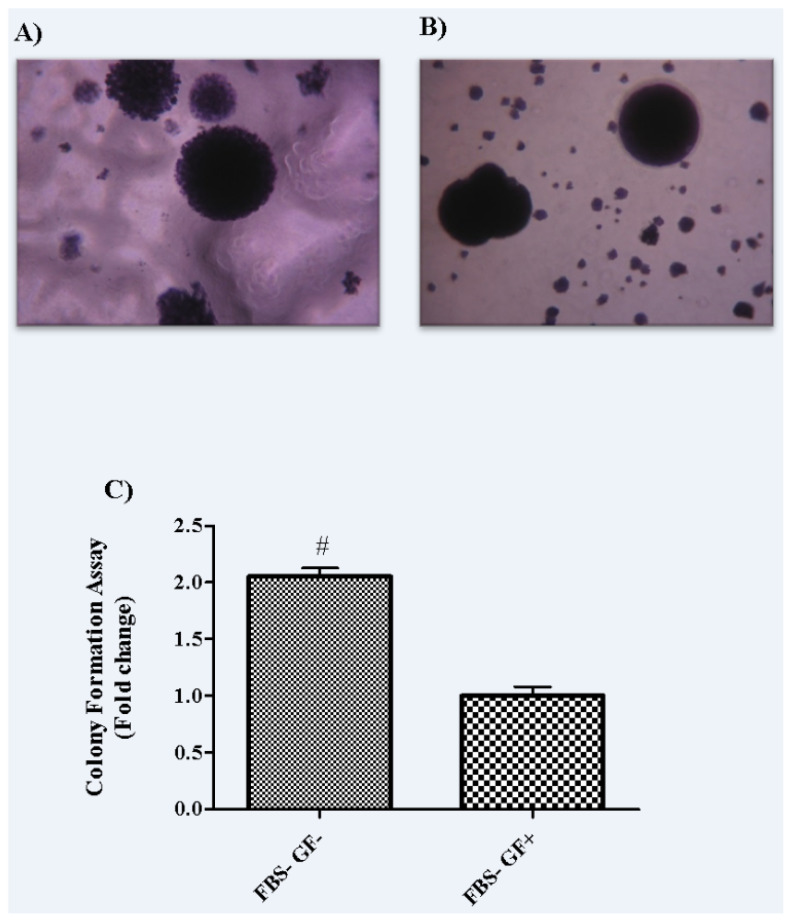
To observe colony formation capacity of spheroid body cells, soft agar assay was performed. A) Group FBS− GF−, B) Group FBS− GF+, C) Fold change of spheroid numbers over Group FBS− GF+. Data are presented as the means ± SD. p < 0.05, comparison of colony-forming capacity. [‘#’ indicates a statistically significant difference compared to Group FBS− GF+]

The group of FBS− GF− had 2 times higher number of colonies when compared to the group of FBS− GF+ (p = 0.02) (Figure 3).

### 3.5. Invasive potential is higher in FBS–GF-free condition than FBS- and GF-positive conditions

**Figure 4:**
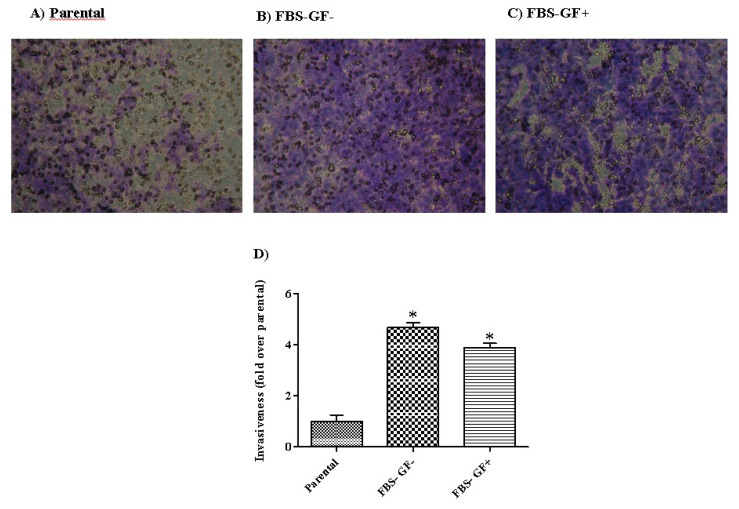
Transwell Matrigel invasion patterns of MKN45 parental, spheroid derived cells after 24 h of plating. A) Parental, B) Group FBS− GF−, C) Group FBS− GF+, D) Invasion rate in spheroid derived and parental cells. ‘*’ indicates a statistically significant difference between parental and spheroid groups (p < 0.05).

The effect of cell culture conditions on invasion capability was determined using the transwell assay. Although each experimental group showed a higher invasive ability compared to the parental cells, the FBS− GF− group exhibited the highest invasive capacity (4.7 fold over parental for FBS− GF− group, 3.9 fold over parental for FBS−GF+ group). Both conditions significantly increased invasive ability compared to parental cells. FBS− GF− group had significantly higher invasion capacity compared to FBS−GF+ group (p = 0.008) (Figure 4).

### 3.6. FBS free condition shows higher chemoresistance against 5-FU

**Figure 5:**
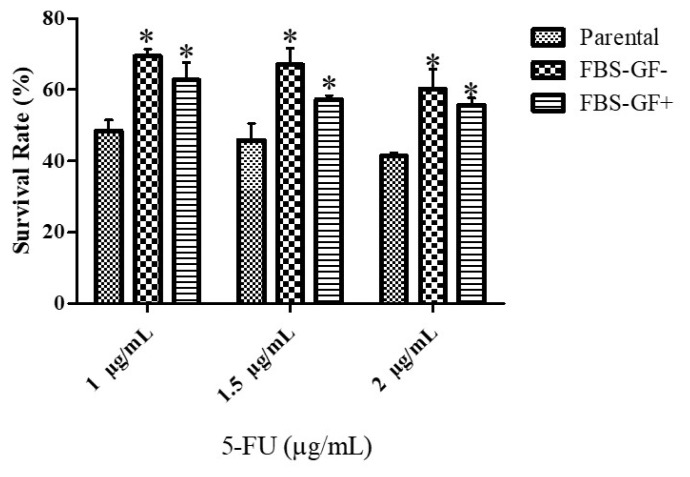
Comparison of chemosensitivity between parental, GF, and FBS–GF-free spheroids of MKN45 after 5-FU treatment for 48 h. 5-FU spheroids. Significant differences in chemosensitivity towards 5-FU were observed between parental cells and Group FBS− GF− spheroid derived cells. ‘*’ indicates a statistically significant difference between parental and spheroid groups (p < 0.05).

The cells were firstly treated with fluorouracil (5-FU), and then the MTS-based cell viability assay was performed to measure the changes in chemoresistance. Spheroids showed variable survival rates after exposure to the drug. Group FBS− GF− had the highest viability against 5-FU for all the doses (Figure 5). FBS− GF− had significantly higher survival rate compared to FBS–GF+ (p = 0.01)

## 4. Discussion

Overexpression of stem cell markers may be related to the self-renewal characteristics of cells. Therefore, we analyzed the stemness-related transcription factors of OCT4 and NANOG and the cell surface markers of MUC1, CD24, CD44, and CD90 for all experimental groups. OCT4 and NANOG, transcription factors, promote mRNA expression of each other synergistically, maintain pluripotency and self-renewal of stem cells, and also control their cell fate (Liu et al., 2013). The CSCs in some types of solid tumors, such as gastric tumors, overexpress OCT4 (Hu et al., 2008; Tang et al., 2011; Liu et al., 2013). NANOG, another CSC marker and an inducer of CSCs properties in several cancers, has a higher expression in cancer stem cells compared to nonstem cancer cells (Lee et al., 2011). MKN45-derived spheroid cells also overexpress both OCT4 and NANOG compared to their adherent counterparts (Liu et al., 2013). Cells can be driven to cell division and differentiation by the same growth factor, such as EGF. Both the duration and intensity of MAP kinase activation determine the cell fate between differentiation or cell division; thus, EGF has a function through this pathway (Günthert et al., 1996). Different growth factors have different functions in the differentiation of several kinds of tissues (Schuldiner et al., 2000). We found that the expression levels of the transcription factors were higher in spheroids than those in adherent cells. On the contrary, growth factor supplementation suppresses the expression levels of some stemness markers in spheroids. Therefore, the potency of cells in the spheroid body may be decreased by GFs-driven differentiation, and this cell differentiation probably caused a decrease in stemness markers expression. However, the effects of GFs on stemness markers were too little compared to the effects of FBS.

We targeted CD90, CD24, and CD44 as CSC surface markers. CD90 is a glycosylphosphatidylinositol (GPI)-anchored glycoprotein and involves cell-matrix and cell-cell interactions (Rege and Hagood 2006). It can be on the surface of different kinds of stem cells, such as mesenchymal stem cells (MSCs), cancer stem cells (CSCs), and in 95% of gastric tumor samples (Cho et al., 2008). CD90 expression is enrichable in serum-free nonadherent spheroid-forming conditions and usable in CSC characterization in primary gastric tumors (Oikonomou et al., 2007; Yang et al., 2008; Jiang et al., 2012). CD90 expression decreased in experimental group supplemented with GFs; therefore, the decrease in CD90 expression in spheroid cells may be an effect of GFs-driven cell differentiation.

CD24, an *O*- and *N*-glycosylated protein, is bound to the extracellular membrane by a glycosylphosphatidylinositol anchor (Pirruccello and LeBien 1986; Fischer et al., 1990). There is a correlation between invasiveness, metastasis, and CD24 expression in gastric cancer (Fujikuni et al., 2014). CD44 is another cell surface transmembrane glycoprotein and a receptor for extracellular matrice ligands, which has roles in cell adhesion, cell-cell interaction, and migration. It is also effective in the initiation and prognosis of stomach cancer (Jang et al., 2011). CD44 positivity may be associated with the maintenance of chemoresistance in MKN-45 and HGC-27 gastric cancer cells (Terzioğlu et al., 2018). CD44 and CD24 are also used to characterize cancer stem cells. CD44+ CD24− cells are more tumorigenic in breast cancer due to their higher invasion and migration ability. However, same characteristics are represented by CD44+ CD24+ cells in gastric cancer (Yan et al., 2013; Fujikuni et al., 2014). Although some previous studies showed increased CD44 levels in spheroid culture with gastric cancer cells, CD44 positivity and expression were lower in spheroid cells in the study. This finding is probably related to the nature of the coating material. Two studies using chitosan and agarose coating for spheroid culture obtained similar results, and they observed a decrease in CD44 expression after a period of spheroid culture (Tsai and Young 2016; Gao et al., 2018). GFs supplementation also decreased the CD44 expression in spheroid cells when the lowest CD24 level was in Group FBS− GF+.

We also measured MUC1 (CD227) expression in spheroid cells. Mucins, structural elements of epithelial cells, have roles in protection, lubrication, and transport. MUC1, an antiadhesive molecule, is overexpressed on the cell surface by breast tumor cells; thus, it inhibits cell adhesion and increases the tumor cells’ metastatic and invasive ability (Lacunza et al., 2010). MUC1 is also an oncogene and supports the maintenance of stemness in embryonic and cancer stem cells (Nath and Mukherjee 2014). MUC1 was upregulated only in Group FBS− GF− spheroids while MUC1 expression decreased in GFs-supplemented spheroids. Therefore, the stemness characteristics of MKN-45 spheroid cells may decrease as a result of GF supplementation (Hikita et al., 2008; Mehla and Singh 2014; Saeki et al., 2014).

Serum-depleted cells are less sensitive to chemotherapeutics, and we found that serum- and GFs-starved cells were more chemoresistant to 5-FU in conjunction with elevated cancer stem cell properties (Cho et al., 2008). The nature of cancer stem cells is known as chemoresistant, and acquired chemoresistance of serum- and GFs-depleted cells may be related to increased expression of stemness markers (Yang et al., 2008). So, indeed, GFs and serum depletion caused an increase in the expression patterns of several stem cell markers, including OCT4, NANOG, MUC1, and CD90. Chemoresistant ovarian cancer cells also have higher expression of cancer stem cell markers, including OCT4 and NANOG (Robinson et al., 2021).Similarly, OCT4 and NANOG expression was upregulated in parallel with increased chemoresistance in FBS− GF− MKN-45 cells. The chemosensitivity of the cells increases after the suppression of MUC1 expression (Deng et al., 2013). Higher MUC1 expression may be another factor related to chemoresistance in MKN-45 cells. CD90+ gastric cancer cells showed a phenotype similar to CSCs after being treated with conventional chemotherapeutics; thus, the percentage of CD90+ gastric cancer cells increased (Fu et al., 2020). Our results indicate that enhanced chemoresistance of FBS− GF− MKN-45 cells from spheroid culture may be due to increased stemness via higher expression of cancer stem cell markers.

Spheroid culture enhances migration capability in pancreatic cancer cell line 1 (PANC-1) while it boosts invasive and metastatic characteristics in glioma cells (Yin et al., 2011; Nonaka et al., 2015). We similarly found that spheroid cells were more invasive in comparison to their parental counterparts. The highest increase in the invasiveness was in FBS− GF− MKN-45 spheroid cells. This finding may be related to the upregulated expression of stemness genes, including OCT4, NANOG, and MUC1, which are effective in invasive capability (Chiou et al., 2010; Retterspitz et al., 2010; Zheng-jie et al., 2012; Lin et al., 2014). Coexpression of OCT4 and NANOG stimulates CSC properties and invasiveness through Stat3/ Snail signaling in hepatocellular carcinoma, while the knockdown of OCT4 decreases invasiveness in pancreatic and gastric cancer cells (Chiou et al., 2010; Zheng-jie et al., 2012; Lin et al., 2014). MUC1 expression is related to invasiveness and metastasis in gastric cancer (Retterspitz et al., 2010; Yonezawa et al., 2012). The relationship between enhanced invasiveness and increased expression of stemness-related genes in GFs- and serum-depleted conditions needs further investigation.

The simultaneous depletion of serum and growth factors could be an important approach for mimicking the tumor environment in cell culture. Comparing the molecular effects of availability or deprivation of growth factors on cultured cancer cells may help find the optimal in vitro cell culture method in which the tumor environment is best mimicked. FBS− GF− spheroids expressed stemness markers in larger amounts compared to FBS− GF+ spheroids. The FBS− GF− medium also caused the formation of higher numbers of spheroids than the FBS− GF+ medium. Therefore, serum- and GF-free mediums can be more favorable for cancer stem cell enrichment and mimicking tumor progression and metastasis in cell culture.

In conclusion, GFs depletion causes an increase in CSC characteristics of MKN-45 cells in terms of increased spheroid body formation, invasion, cancer stem cell markers, and chemoresistance. Although EGF and FGF-2 are commonly used in spheroid cell culture, it may not be necessary for MKN-45 spheroid cell culture according to our findings on the usage of RPMI-1640 medium without GFs and FBS. EGF and FGF-2 seem to cause different outcomes; therefore, further analysis should be performed to explain the mechanism of this paradoxical effect of GFs depletion.

## Figures and Tables

**Table 1:** Names and the contents of the experimental groups.

Name of the experimental group	Content
FBS− GF−	Culture medium contains no FBS or GFs
FBS− GF+	Culture medium contains 20 ng/mL human FGF-2, 20 ng/mL EGF, but no FBS

**Table 2:** List of the used TaqMan gene expression assays.

TaqMan gene expression assay	Product number
OCT4	Hs04260367_gH
NANOG	Hs04260366_g1
CD24	Hs02379687_s1
CD90	Hs00264235_s1
MUC1	Hs00159357_m1
SOX2	Hs00602736_s1
BETA ACTIN	Hs99999903_m1
